# Persistent Hypotony Following Antiglaucoma Surgery: A Case Report

**DOI:** 10.1155/crop/8839203

**Published:** 2025-07-11

**Authors:** Shuxin Cai, Haibo Li, Shuimiao Chen, Ranqing Lin

**Affiliations:** ^1^Ocular Trauma Department, Eye Institute and Affiliated Xiamen Eye Center of Xiamen University, School of Medicine, Xiamen University, Xiamen, China; ^2^Xiamen University, Xiamen, Fujian, China; ^3^Fujian Provincial Key Laboratory of Corneal & Ocular Surface Diseases, Xiamen, Fujian, China; ^4^Xiamen Municipal Key Laboratory of Corneal & Ocular Surface Diseases, Xiamen, Fujian, China; ^5^Xiamen Research Center for Eye Diseases and Key Laboratory of Ophthalmology, Xiamen, Fujian, China

**Keywords:** antiglaucoma surgery, cataract, persist hypotony

## Abstract

**Background:** Persistent hypotony is a rare but serious complication following antiglaucoma surgery. Identifying the cause and appropriate management is critical to prevent vision loss.

**Case Presentation:** A 56-year-old male presented with 6 months of progressive vision loss in the left eye, 20 years post-antiglaucoma surgery. Examination showed no light perception in the right eye and choroidal and ciliary body detachment with lens subluxation in the left eye. After a month of conservative treatment, intraocular pressure (IOP) in the left eye remained below 6 mmHg, necessitating surgical intervention. Combined suprachoroidal fluid drainage, phacoemulsification, and capsular tension ring implantation improved visual acuity and stabilized IOP. Six months postoperatively, the best corrected visual acuity (BCVA) was 0.3.

**Conclusions:** Prompt identification and treatment of persistent hypotony post-antiglaucoma surgery are essential for visual function restoration and complication prevention.

## 1. Background

Hypotony, defined as IOP below 6 mmHg, is a common complication postglaucoma surgery due to increased aqueous outflow or insufficient aqueous production. This may result in hypotonus maculopathy, optic disk edema, corneal folds, and astigmatism, which may ultimately lead to vision loss [1]. It is therefore of the utmost importance to promptly identify the cause and initiate appropriate management. This report describes the treatment of a patient with long-term hypotony. In the case of a patient presenting with vision loss in one eye, it is of the utmost importance to accurately identify the cause of hypotony and implement appropriate management in order to prevent the occurrence of unnecessary complications.

## 2. Case Presentation

**Figure 1:**
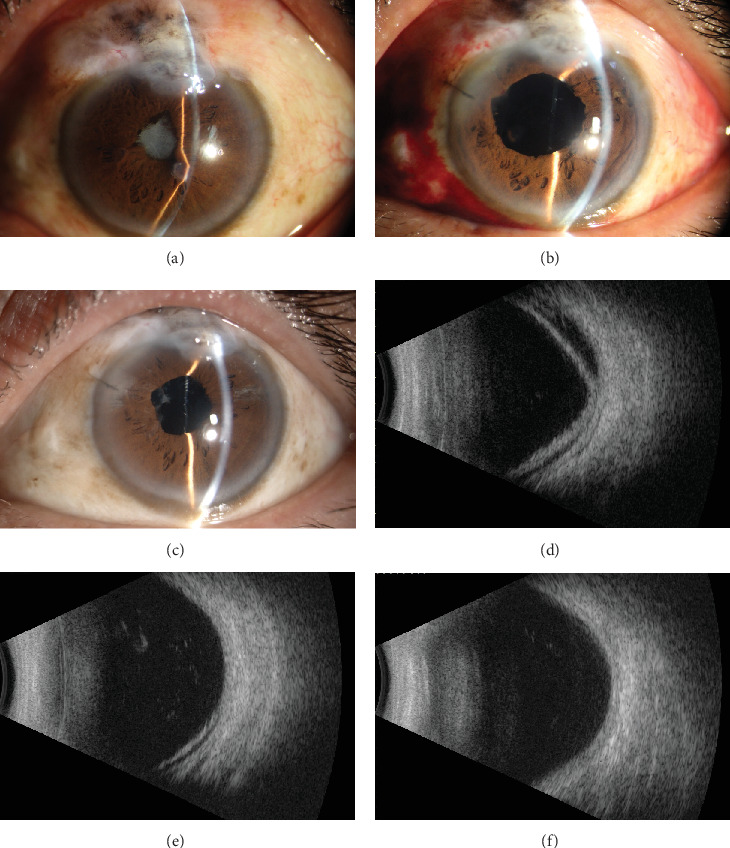
Slit lamp photography and B-scan of the left eye with prolonged low intraocular pressure after antiglaucoma surgery. (a) Preoperative slit lamp photography showing pupil irregularity and cataract. (b) Slit lamp photography showing an enlarged pupil 1 day postsurgery. (c) Slit lamp photography showing a regular pupil 1 month postsurgery. (d) B-scan showing an extensive choroidal detachment before operation. (e) B-scan showing a local choroidal detachment 1 day postsurgery. (f) B-scan showing no choroidal detachment 1 month postsurgery.

**Figure 2:**
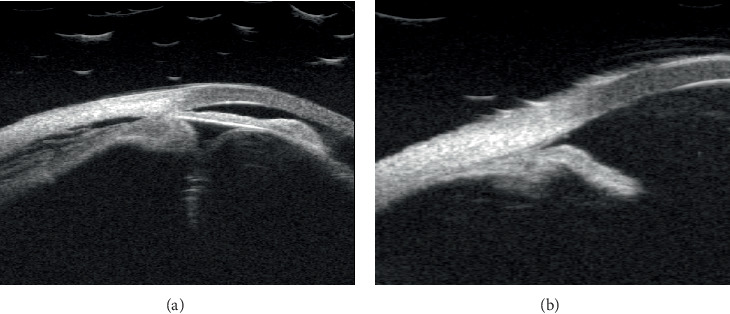
UBM examination photography in patient with long-term low intraocular pressure after surgery. (a) UBM showed circumferential ciliary body detachment and anterior and posterior iris adhesions before surgery. (b) UBM showed no circumferential ciliary body detachment and choroidal detachment 6 months postsurgery.

**Figure 3:**
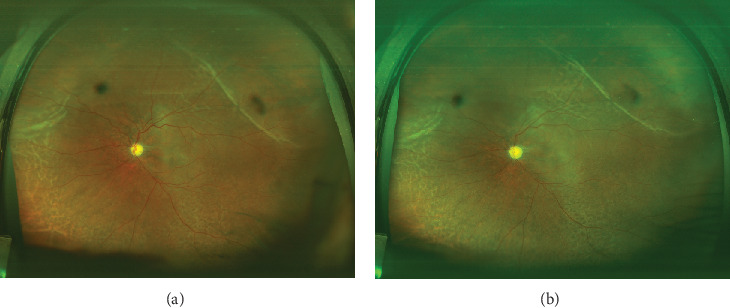
Fundus examination photography in a patient with long-term low intraocular pressure after surgery. (a) Fundus photograph showed a pale papilla and choroidal folds in the supranasal and supratemporal sites 1 day postsurgery. (b) Fundus photograph showed a pale papilla and choroidal folds in the supranasal and supratemporal sites that still exist 6 months postsurgery.

**Figure 4:**
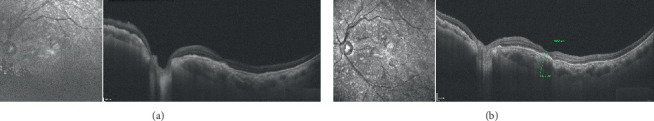
OCT in patient with long-term low intraocular pressure after surgery. (a) OCT showing strong sublayer neuroepithelial reflex and irregular RPE 1 day postsurgery. (b) OCT showing a foveal thickness of 189.7 *μ*m and strong sublayer reflex 6 months postsurgery.

A 56-year-old male presented with recent vision decline in the left eye, previously diagnosed with primary angle-closure glaucoma and treated with trabeculectomy 20 years prior. The patient had been blind in the right eye for 10 years due to uncontrolled IOP, with no light perception. Examination revealed an IOP of 19.0 mmHg in the right eye and 5.9 mmHg in the left eye. He denied the history of uveitis. A slit lamp examination of the anterior segment revealed a shallow anterior chamber in both eyes and a flattened filtering bleb in the right eye. In the left eye, the filtering bleb exhibited fibrosis, pigment deposition, posterior synechiae, and pupillary occlusion (Figure 1). B-scan imaging demonstrated the presence of a choroidal detachment, and the axial length of 21.4 mm was obtained by B-scan (Figure 1), while ultrasound biomicroscopy (UBM) revealed the presence of circumferential ciliary body detachment and lens subluxation in the left eye (Figure 2). After topical and systemic steroids were administered for 1 month, the hypotony still persisted. The IOP in the left eye remained below 6 mmHg, and surgical intervention was deemed necessary. Under local anesthesia, a combined surgical approach was employed to address the patient's left eye pathology, consisting of transscleral drainage for choroidal effusion, phacoemulsification cataract extraction, and capsular tension ring (CTR) implantation. The patient, presenting with choroidal effusion secondary to ocular pathology and concomitant cataract, required a multifaceted surgical intervention to manage both conditions simultaneously. The transscleral drainage procedure is aimed at alleviating the choroidal effusion by creating a controlled drainage pathway through the sclera, which was anticipated to reduce IOP and mitigate the risk of further retinal or optic nerve damage. Phacoemulsification was performed to remove the opacified lens material, followed by CTR implantation to provide structural support to the lens capsule, ensuring its stability and reducing the risk of posterior capsule rupture or zonular dehiscence (see Video S1, Supporting Digital Content 1). During the operation, staining was used to examine the filtration bleb, which demonstrated that the bleb was not functional. One day after the surgery, slit lamp photography showed an enlarged pupil (Figure 1); fundus photograph revealed no evidence of retinal tears but a pale papilla and choroidal folds in the supranasal and supratemporal sites (Figure 3). The IOP was increased to 9.5 mmHg, and best corrected visual acuity (BCVA) was increased to 0.2. The B-scan ultrasound examination showed a slight detachment of the temporal quadrant choroid in the left eye (Figure 1), with the OCT showing strong sublayer neuroepithelial reflex and irregular RPE (Figure 4). Four weeks after the initial examination, the IOP remained at 12.1 mmHg, while the BCVA was 0.25. The B-scan ultrasound examination demonstrated no evidence of detachment of the choroid (Figure 1). Six months later, the pupil became regular (Figure 1), the IOP remained at 10.8 mmHg, and the OCT showed a foveal thickness of 189.7 *μ*m and strong sublayer reflex (Figure 4), while the BCVA had improved to 0.3.

## 3. Discussion

Hypotony and choroidal detachment are common complications observed in patients who have undergone antiglaucoma surgery. Transient hypotony does not typically result in permanent visual impairment; however, persistent hypotony can lead to ocular hypoperfusion, impaired aqueous circulation, tissue hypoxia, and nutrient deprivation. This can result in tissue edema, blood-aqueous barrier disruption, increased inflammatory exudation, and altered aqueous composition and metabolism, leading to permanent ocular damage [1]. Hypotony may also result in the leakage of serum from the ciliary body and choroidal vessels into the suprachoroidal space, which can lead to an elevation of the choroid [2] and subsequent ciliary body and choroidal detachment. Additionally, it may lead to complications such as secondary cataract, corneal endothelial decompensation, and severe visual loss [3].

Following antiglaucoma surgery, two main categories of hypotony are commonly observed as a result of the procedure. Firstly, there is an increased aqueous outflow, which may be due to excessive filtration bleb function, bleb leakage, choroidal and ciliary body detachment, retinal holes, or retinal detachment [4]. Secondly, inadequate aqueous secretion may result from a number of factors, including toxic effects of antimetabolite drugs, which can lead to ciliary body dysfunction. Similarly, chronic uveitis can cause impairment of ciliary body function [5]. The patient underwent antiglaucoma surgery 20 years ago. Ten years later, he was diagnosed with elevated intraocular pressure in both eyes during a visit to an ophthalmologist due to right-eye blindness. The long-term topical use of intraocular pressure-lowering medications effectively controlled the intraocular pressure, thus ruling out long-term hypotony caused by excessive filtration from the initial antiglaucoma surgery. Furthermore, intraoperative exploration revealed the absence of filtration function in the filtration bleb. Based on the medical history and clinical presentation, it is postulated that the prolonged low intraocular pressure may be associated with chronic uveitis.

Hypotony represents a rare complication of uveitis and is closely associated with vision impairment. The risk factors include the duration of uveitis and the presence of severe complications such as iris adhesions [6]. Upon slit lamp photographs preoperatively (Figure 1), we hypothesized that the abnormal wound healing following antiglaucoma surgery and postoperative conjunctival pigment exposure may result in the occurrence of uveitis, which in turn impairs the secretory function of the ciliary epithelium. This ultimately leads to reduced aqueous humor secretion and, consequently, low IOP [7]. Due to the individual variations in patient tolerance to hypotony, as well as differences in the duration and degree of hypotony, the extent of visual impairment among patients will also vary. Preoperative UBM revealed circumferential ciliary body detachment and anterior and posterior iris adhesions (Figure 2). During the outpatient treatment period, the IOP remained below 6 mmHg for an extended period. Concomitant with B-scan ultrasonography, the diagnosis is low intraocular pressure syndrome accompanied by chronic uveitis.

In this case, intraoperatively, the drainage of suprachoroidal fluid was performed in conjunction with the release of iris adhesions and the implantation of a tension ring. Subsequently, the ciliary body and choroid were repositioned, and IOP gradually recovered, accompanied by a gradual improvement in vision. At the 6-month follow-up, the patient's intraocular pressure remained stable, with BCVA maintained at 0.3, and there was no recurrence of ciliary body detachment (Figure 2), choroidal detachment (Figure 3), or uveitis. In this case, refractory hypotony was successfully treated by addressing multiple factors, including intraoperative simultaneous correction of lens dislocation, ciliary body, and choroidal detachment, along with one-stage implantation of a CTR. This approach partially restored normal anterior segment tissue anatomy and allowed for the normalization of aqueous humor circulation and metabolism. Given the patient's short axial length, the potential errors in measuring the power of the intraocular lens, and the risk of recurrent uveitis, it was decided that an intraocular lens would not be implanted during the initial surgery. Subsequent to the surgical procedure, visual acuity was enhanced through the use of corrective lenses.

## 4. Conclusions

Timely identification and management of persistent hypotony post-antiglaucoma surgery are crucial for visual rehabilitation. Surgical intervention addressing the underlying causes can result in favorable outcomes.

## Data Availability

The data that support the findings of this study are available from the corresponding author upon reasonable request.

## Supporting Information

Supporting Information Additional supporting information can be found online in the Supporting Information section. Video S1: The supporting video demonstrates the key surgical steps of combined suprachoroidal fluid drainage, phacoemulsification, and capsular tension ring implantation.
